# Effects of age on metacognitive efficiency

**DOI:** 10.1016/j.concog.2014.06.007

**Published:** 2014-08

**Authors:** Emma C. Palmer, Anthony S. David, Stephen M. Fleming

**Affiliations:** a Institute of Psychiatry, King’s College, London, UK; b Center for Neural Science, NYU, United States; c Department of Experimental Psychology, University of Oxford, UK

**Keywords:** Metacognition, Aging, Lifespan, Development, Awareness

## Abstract

Humans have a capacity to become aware of thoughts and behaviours known as metacognition. Metacognitive efficiency refers to the relationship between subjective reports and objective behaviour. Understanding how this efficiency changes as we age is important because poor metacognition can lead to negative consequences, such as believing one is a good driver despite a recent spate of accidents. We quantified metacognition in two cognitive domains, perception and memory, in healthy adults between 18 and 84 years old, employing measures that dissociate objective task performance from metacognitive efficiency. We identified a marked decrease in perceptual metacognitive efficiency with age and a non-significant decrease in memory metacognitive efficiency. No significant relationship was identified between executive function and metacognition in either domain. Annual decline in metacognitive efficiency after controlling for executive function was ∼0.6%. Decreases in metacognitive efficiency may explain why dissociations between behaviour and beliefs become more marked as we age.

## Highlights

• Metacognitive ability refers to the mapping between one’s beliefs and behaviours.
• We separately quantified task performance and metacognition for perception and memory.
• We identify a decline in metacognition with age across the adult lifespan.
• Changes of metacognition with age were not explained by executive function.
• Combined with adolescent data the lifespan profile of metacognition is an inverted-U.

## 1 Introduction

Metacognition refers to ‘thinking about thinking’ (Flavell, 1979), or the ability to become aware of thoughts and behaviours. Metacognition is a fundamental aspect of higher cognition in humans (Dunlosky & Metcalfe, 2009) and may support conscious awareness (Koriat, 2007), social interaction (Frith, 2012), and be impaired in neuropsychiatric disorders (David, Bedford, Wiffen, & Gilleen, 2012). Metacognition comprises both monitoring and control (Koriat and Goldsmith, 1996, Nelson, 1996). Intuitively, a person has good metacognitive monitoring if their subjective appraisals track their objective behaviour on a trial-by-trial basis. For example, an individual who is metacognitively efficient will report high confidence when they are objectively correct, and low confidence when they are objectively incorrect; conversely an individual with poor metacognitive efficiency has poor awareness, manifested by subjective reports that are unrelated to task performance.

Different cognitive domains give rise to different types of metacognitive judgment. For example, in the perceptual domain one might have variable confidence about seeing or hearing a particular stimulus. Metacognitive judgments about memory may refer to whether one’s recall or recognition is likely to have been correct (a retrospective confidence rating) or whether a stimulus is likely to be remembered or recognised in the future (“judgments of learning”, JOLs; and “feelings of knowing”, FOK). Overall, metacognitive judgments are thought to be based on a combination of perceptual or mnemonic strength and additional analytic factors (Busey et al., 2000, Koriat and Goldsmith, 1996, Vickers, 1979). Previous research has established that metacognitive efficiency in different domains can be isolated and studied independently of primary cognitive capacity (see Fleming & Dolan, 2012, for a review).

However, there is some debate as to whether metacognition changes as we age. On the one hand, we might expect greater life experience leads to more accurate self-knowledge and greater metacognitive efficiency. On the other hand, convergent evidence has revealed a specific neural basis for metacognitive efficiency in human prefrontal and parietal cortex (Fleming et al., 2012, Fleming et al., 2010, McCurdy et al., 2013, Rounis et al., 2010, Yokoyama et al., 2010) regions which are also highly susceptible to aging-related atrophy (Raz et al., 2005, Resnick et al., 2003) and therefore metacognitive efficiency may be expected to decrease as we age. Such a hypothesis is consistent with reports that lack of awareness of cognitive, physical and perceptual abilities in healthy older adults can be problematic in everyday life. (Hertzog & Hultsch, 2000) demonstrated that there are notable changes in self-appraisal as we age, and these tend to centre on inaccuracies regarding beliefs about cognitive ability and control over cognition. Older adults tend to demonstrate increased over-confidence compared to actual performance when compared to younger adults (Dodson et al., 2007, Hansson et al., 2008). For example when older adults between the ages of 65–91 years old were asked about their driving abilities, 85% of the drivers in this age range rated themselves as ‘good’ or ‘excellent’ drivers despite an increased frequency of accidents (Ross, Dodson, Edwards, Ackerman, & Ball, 2012).

However, the literature on laboratory measures of metacognition such as confidence judgments and JOLs has shown mixed results. Some studies reveal stable or even improved accuracy of confidence ratings with age for general knowledge (Dodson et al., 2007, Pliske and Mutter, 1996), problem solving (Vukman, 2005), or memory recall tasks (Lachman, Lachman, & Thronesbery, 1979). Similarly, studies investigating JOLs, FOKs and “judgments of forgetting” have found that older adults’ predictions of recall or recognition were as good as those of younger adults (Eakin et al., 2014, Haber, 2012, Halamish et al., 2011). In contrast, other studies report significant age differences in the accuracy of confidence judgments about recall and recognition (Bender and Raz, 2012, Dodson et al., 2007, Huff et al., 2011, Kelley and Sahakyan, 2003, Pansky et al., 2009, Perrotin et al., 2006, Soderstrom et al., 2012, Souchay et al., 2000, Souchay et al., 2007, Toth et al., 2011, Wong et al., 2012) learning of emotional information (Tauber & Dunlosky, 2012), and study-time allocation (Froger, Sacher, Gaudouen, Isingrini, & Taconnat, 2011). In addition, the neural correlates of metacognitive judgments have been found to differ between younger and older adults (Chua, Schacter, & Sperling, 2009).

In many of these studies it has proven difficult to decouple metacognitive accuracy from age-related changes in performance. Common measures of metacognitive accuracy such as the gamma correlation are affected by task performance (Masson & Rotello, 2009), potentially confounding changes in metacognition with age with changes in performance. For example, if two individuals A and B have identical metacognitive ability, but A performs better than B on the primary task, A’s metacognition score will appear higher than B’s due to this performance confound. Accordingly, Daniels, Toth, and Hertzog (2009) found that older adults had lower accuracy of immediate JOLs for predicting old/new item recognition, but reasoned that this may reflect age-related memory deficits as opposed to deficits in metacognition. In the present study we employ a recently developed signal detection theoretic measure, meta-d′/d′ (Maniscalco & Lau, 2012), to circumvent this problem. Meta-d′/d′ quantifies the efficiency with which confidence ratings discriminate between correct and incorrect trials in each task domain (perception and memory). Importantly, meta-d′/d′ is a relative measure: given a certain level of processing capacity (d′), meta-d′/d′ quantifies the extent to which a metacognitively optimal observer is aware of their performance.

Previous literature has also drawn conceptual similarities between characteristics of memory metacognition and executive functions (Fernandez-Duque et al., 2000, Pannu and Kaszniak, 2005, Shimamura, 1995, Souchay et al., 2000). In particular it has been suggested that any age-related decline in metacognition may be due to executive limitations associated with aging (Souchay & Isingrini, 2004). Again, however, results from initial studies examining this issue are mixed. FOK but not JOL accuracy has been shown to significantly correlate with executive function (Souchay, Isingrini, Clarys, Taconnat, & Eustache, 2004), indicating that perhaps only some forms of metacognitive judgement require intact executive function to be completed accurately. Perrotin, Belleville, and Isingrini (2007) compared patients with mild cognitive impairment (MCI) to healthy age-matched controls in their FOK abilities. FOK accuracy was primarily related to primary memory performance in MCI patients, whereas in control participants it was linked to executive function.

In the present study we therefore examined the effects of both age and executive function on a performance-controlled measure of metacognitive efficiency (meta-d′/d′). We studied retrospective confidence judgments in two domains, perception and memory, following recent evidence that metacognition across different domains may draw upon dissociable neural and cognitive processes (Baird et al., 2013, McCurdy et al., 2013).

## 2 Materials and methods

### 2.1 Recruitment

Participants were recruited via two distinct sampling methods as part of a larger clinical research study. A copy of the Royal Mail Postal Address File (PAF), containing all residential addresses in the boroughs of Lambeth and Southwark (London, UK), was obtained, and approach letters for participation in the study were sent out to a random selection of 184 addresses. A total of 28 participants were recruited using this method. Participants verbally confirmed an absence of mild cognitive impairment (MCI) or memory disorder.

Participants were also recruited using the MindSearch database, available to researchers based at King’s College, London. Participants who had registered their details with the database and met the inclusion criteria (*n* = 585) for this study were selected. All potential participants were contacted via email twice in a 6 month period, with a total of 32 participants responding positively and being included in the study. Our inclusion criteria required participants to be over the age of 18 and have no previous or current presentation of a psychosis related disorder, MCI, memory disorders or Alzheimer’s dementia.

We compared our healthy older adult sample to a sample of confirmed MCI patients who were screened as part of a larger clinical study (criteria = scoring > 20 on the MMSE and failing at least one section of the Consortium to Establish a Registry for Alzheimer’s Disease (CERAD) neuropsychological battery). The PAF and MindSearch participants performed significantly better on the Wechsler Memory Scale (immediate and long term recall, long term recognition) and Trails B-A executive function test than the clinical sample. Therefore we can be confident that the majority of the participants included were not suffering from undiagnosed MCI.

There was no significant difference between the two recruitment methods regarding mean group age (*t* = .27, *p* = .79), years in education (*t* = −.49, *p* = .63) or BDI score (*t* = −.52, *p* = .61). The sample was recruited using opportunity sampling within the two methods with no set age/gender cells filled. Two subjects with Beck Depression Inventory (BDI; (Beck & Steer, 1984) scores greater than 20 were excluded from further analysis. All participants had normal or corrected to normal vision.

### 2.2 Participants

Our sample consisted of 60 participants, 33 of whom were female. The mean age was 40.28 years old, with a minimum of 18 and maximum of 84 years old, with no difference in age between males and females (*t*(58) = 0.76, *p* = 0.45). The mean depression score was 6.03 (SD = 4.32) out of a possible 64 indicating that the sample was not depressed. A subset of participants completed the memory metacognition and neuropsychological tests; therefore for each test the relevant *N* is stated (Table 1).

**Table 1:** Characteristics of participants and experimental measures together with their relationship with age. (^∗^*P* < 0.05; ^∗∗^*P* < 0.01.)

	Measure	*N*	Mean (SD)	Range	Corr. with age (Pearson’s *r*)	*P*-value
Participant characteristics	BDI	60	6.03 (4.32)	0–20	−0.08	0.54
	Years of education	60	14.6 (2.5)	7–17	−0.37	0.003⁎⁎

Neuropsychological tests	WAIS-III IQ	39	115.1 (15.9)	76–146	−0.28	0.08
	Trails B-A	44	30.5 (20.4)	11.3–99.1	0.20	0.18
	Wechsler Memory Scale (age-adjusted)					
	Immediate	34	11.2 (3.67)	14	−.036	.839
	Delayed	34	12.1 (3.69)	13	−.025	.892
	Recognition	33	26.6 (3.68)	19	−.302	.088

Metacognitive accuracy	Perceptual meta-d′/d′	53	1.08 (0.34)	0.40–1.96	−0.38	0.0051⁎⁎
	Memory meta-d′/d′	38	0.73 (0.81)	−2.25–1.85	−0.064	0.70

^∗^ *P* < 0.05.

⁎⁎ *P* < 0.01.

### 2.3 Metacognition measures

**Fig. 1:**
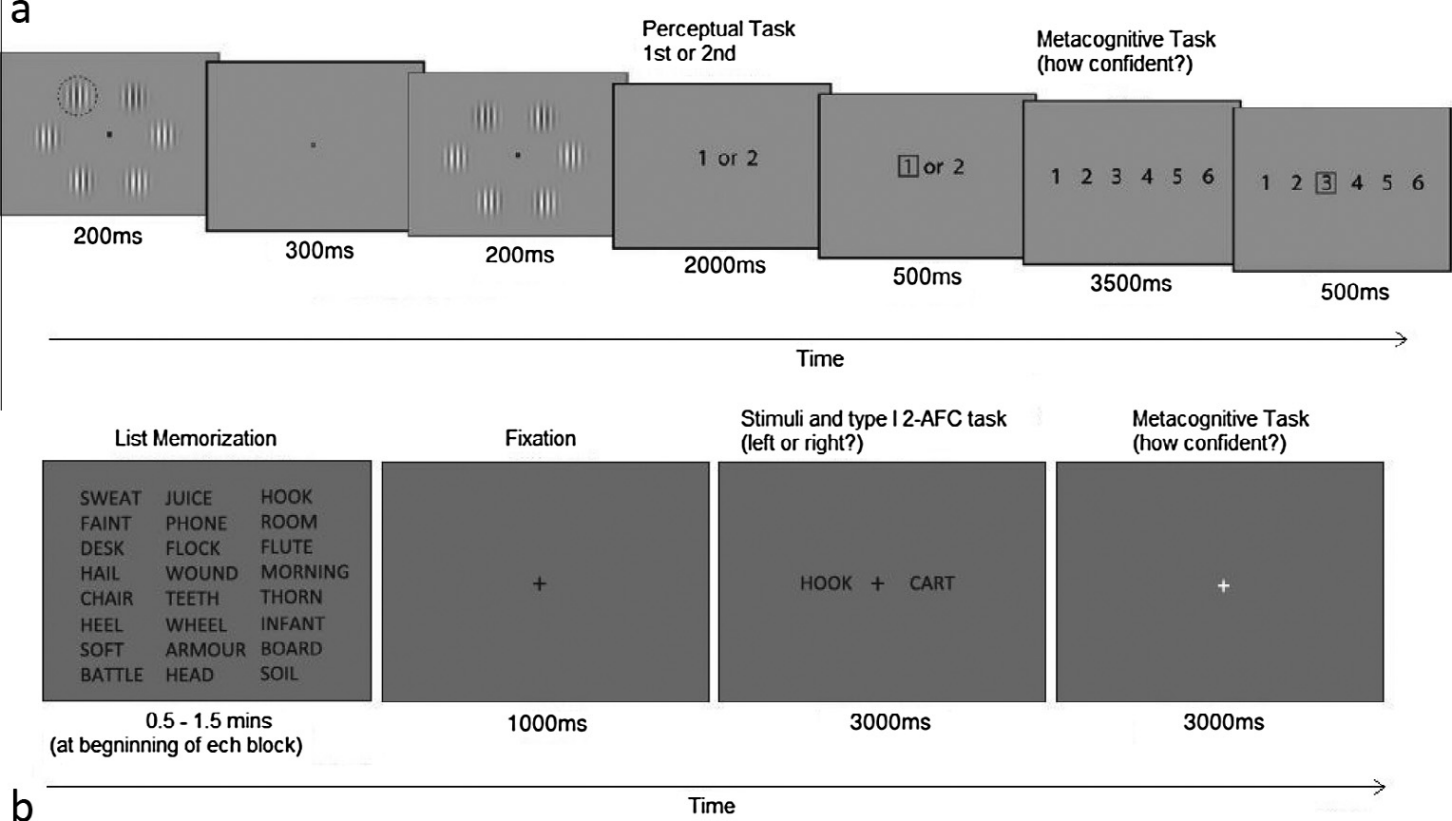
(a) Both the perceptual and memory tasks required two judgments per trial: a two-alternative forced-choice perceptual/mnemonic decision followed by an estimate of relative confidence in each decision. (a) Perception task. Participants responded as to which interval (first, second) contained the “pop out” grating stimulus and then rated confidence in their decision. (b) Memory task. At the beginning of each block, participants studied a list of 50 words arranged in 10 rows and 5 columns (a 8 row × 3 column example is shown here for display purposes). On each trial participants were asked to indicate which word (left or right) was on the previously studied list and to subsequently rate their confidence in their decision.

*Perceptual metacognitive ability* was investigated using a computerised visual perceptual task similar to that used previously (Fleming et al., 2010). Each trial required participants to perform a perceptual task. The stimuli used were Gabor patches: circular patches of alternating light and dark vertical bars (2.8 visual degrees in diameter, spatial frequency of 2.2 cycles per visual degree). The contrast between the vertical lines in each standard Gabor patch was 20%, where 0% indicates no difference between the light and dark bars and 100%, the maximum difference (black to white). Six such Gabor patches were arranged in a circle (eccentricity of 6.9 visual degrees) around a central fixation point set on a uniform grey background (see Fig. 1a). One of the six Gabor patches was made to pop-out from the others by increasing the contrast in that patch. The contrast of the pop-out Gabor patches varied from 23% (little effect of pop-out) to 80% (pop-out very clear). The task required participants to view two stimulus arrays, each presented for 200 ms, separated by an interval of 300 ms. The interval between stimuli was filled by a uniform grey screen without the Gabor patches. A single Gabor patch in one of the two intervals was designated as a pop-out. Which of the six Gabor patches popped-out varied randomly between trials.

Participants were prompted by a computer display to respond ‘1’ or ‘2’ as to whether they thought the pop-out Gabor patch appeared during the first or second presentation. Participants responded by pressing the numerical keys on the top left-hand side of the laptop keyboard with their left hand. Participants had 2 s in which to make their decision, after which a red box surrounded their selection. No feedback was given as to whether they were right or wrong.

Participants then indicated confidence in their decision on a scale of 1–6 (1: relatively low confidence; 6: relatively high confidence; see Fig. 1a.). Participants were encouraged to use the full range of the scale, thinking carefully about how confident they were after each decision. Participants responded by pressing numerically marked keys on the top left-hand side of the laptop keyboard with their left hand, with a red box again surrounding this selection. Participants had 3.5 s to complete this metacognitive judgement. Performance on the task was maintained at around 70% using a 2-down, 1-up staircase procedure (Levitt, 1971). Two consecutive correct visual judgments led to a one step (3%) decrease in contrast of the pop-out Gabor patch in the next trial, whereas one incorrect visual judgment led to a one step increase in contrast of the pop-out patch. This procedure ensures all participants perform with approximately the same accuracy on the primary perceptual task, allowing us to measure metacognitive ability independent of task performance. This was especially useful in the present study as the range of participant ages may otherwise have led to performance bias. Older participants who struggled to make manual responses gave verbal answers to a researcher who made manual responses. The task comprised 5 blocks of 8 min with short breaks between each block, taking approximately 50 min to complete.

A standard task instruction sheet was read through by participants on their own, followed by the opportunity to ask the task administrator questions. Participants were seated in a darkened room approximately 60 cm from a laptop computer screen (Sony Vaio, PCG-71614 M laptop; 17 in display; 1280 × 800 pixels). Stimulus display and responses for the tasks were programmed in MATLAB 7.8 (Mathworks Inc., Natick, MA, USA) using the COGENT 2000 toolbox (http://www.vislab.ucl.ac.uk/cogent.php). A practice session of two blocks of eight trials was given at the start to familiarise participants with the task. Participants were tested individually in a quiet room.

*Memory metacognitive ability* was investigated using the 2-alternative forced choice (AFC) memory confidence task devised by McCurdy et al. (2013) (see Fig. 1b). There were three learning and testing blocks, with a different learning time assigned to each block. At the beginning of each block, 50 English words (Calibri font, size 24) were presented simultaneously on the screen for either 0.5, 1, or 1.5 min to create three levels of difficulty in which participants performed at neither chance nor ceiling. English words were generated using the Medical Research Council Psycholinguistic Database (Wilson, 1988). These standard nouns were four to eight letters long, had one to three syllables, and had a familiarity, concreteness, and imagability rating of 400–700 each. Participants were instructed to memorize as many words on the list as possible during the study period. A small notice appeared at the bottom of the screen to inform them when there was 10 s left to study the list. After the study period, a series of trials probing memory for the word list was presented. In each trial, two words were presented to the left and right of fixation. One of these words had been presented on the study list (“old”), and the other word had not been presented previously (“new”). First, participants had 3s to provide a 2-AFC judgment with regard to which word was ‘old’, where pressing ‘1’ referred to the left hand word and ‘2’ referred to the right hand word. Participants then had 3 s to press one of four keys (“7,” “8,” “9,” or “0”) using their right hand to indicate their confidence in being correct on the 2-AFC judgment (signifying “not at all confident” to “very confident” respectively). Older participants who struggled to make manual responses gave verbal answers to a researcher who made manual responses. The task comprised 3 blocks of approximately 5 min each with short breaks in between each block.

#### 2.3.1 Calculating metacognitive efficiency

Metacognitive efficiency was quantified using the meta-d′ measure developed by Maniscalco and Lau (2012). Meta-d′/d′ is a relative measure: given a certain level of processing capacity (d′), meta-d′/d′ quantifies the extent to which a metacognitively optimal observer is aware of their performance. A meta-d′/d′ value of 1 is equivalent to metacognitively “ideal”, whereas meta-d′/d′ < 1 indicates a failure of metacognitive awareness. Values of meta-d′/d′ greater than 1 indicate that awareness is more accurate than task performance, which may occur for instance if the initial judgment is made under time pressure (Charles, Opstal, Marti, & Dehaene, 2013). Meta-d′ was calculated using MATLAB code available at http://www.columbia.edu/~bsm2105/type2sdt (Maniscalco & Lau, 2012) for both perceptual and memory metacognition.

### 2.4 Neuropsychological measures

IQ was measured using the shortened Wechsler Adult Intelligence Scale-III (WAIS-III; Wechsler, 1997a, Wechsler, 1997b) which comprises the Digit Symbol Coding, Arithmetic, Information and Block Design sub-components of the full WAIS assessment; 39 participants completed this assessment.

Executive function (EF) was measured using the “Trail Making” test (Reitan, 1986), which measures ‘set-shifting’, or the ability to shift attention between one task to another. This test consists of two parts in which the subject is instructed to connect a set of 25 dots as fast as possible while still maintaining accuracy. The first task (A) requires participants to connect numbers in ascending order and the second task (B) requires participants to connect the dots, alternating between numbers and letters (in alphabetical order). Time taken to complete both tasks is recorded and a ‘B-A’ score is calculated by subtracting the time taken in task A from time taken in task B. The longer the composite time (B-A) the worse the set-shifting ability. 44 participants completed this assessment.

The Wechsler Memory Scale (WMS) ‘logical memory’ sub-task (Wechsler, 1997a, Wechsler, 1997b) was used to gauge short-term, long-term and recognition memory. The logical memory task is a sub-test within the whole WMS battery, designed to detect attention and memory deficits, and asks participants to listen to and remember two spoken stories. After hearing each story participants are asked to immediately recount the story to the researcher (Immediate test), where a higher score is obtained by recalling more key facts. 30 min after the stories are initially heard participants are asked again to recount the stories to the researcher (Delayed test). Finally, a recognition task is then carried out, where participants are asked 15 questions about each story and required to give ‘Yes’ or ‘No’ answers (Recognition test). 34 participants completed the short-term recall (Immediate) test, and 33 participants completed the long-term recall (Delayed) and recognition (Recognition) tests.

### 2.5 Data analysis

Correlations between metacognitive efficiency (meta-d′/d′), age and neuropsychological measures were computed using Pearson’s product-moment correlations implemented in R 3.0.1. 5 subjects who performed lower than 65% in the perceptual task (indicating a failure of the staircase procedure to appropriately control performance) were excluded from analyses of perceptual task data. Regression analyses were carried out using the lm function in R and un-standardised regression coefficients are reported.

## 3 Results

The mean percentage of trials answered correctly on the perceptual task was 70.8% (range 65.7–73.6%), with no significant relationship between age and percentage of trials answered correctly (*r* = −0.16, *p* = 0.25; *n* = 53). The mean percentage of trials answered correctly on the memory task was 67.8% (range 49–87%). Performance on the memory task was lower in older adults leading to a negative correlation between age and performance (*r* = −0.36, *p* = 0.03; *n* = 38).

**Fig. 2:**
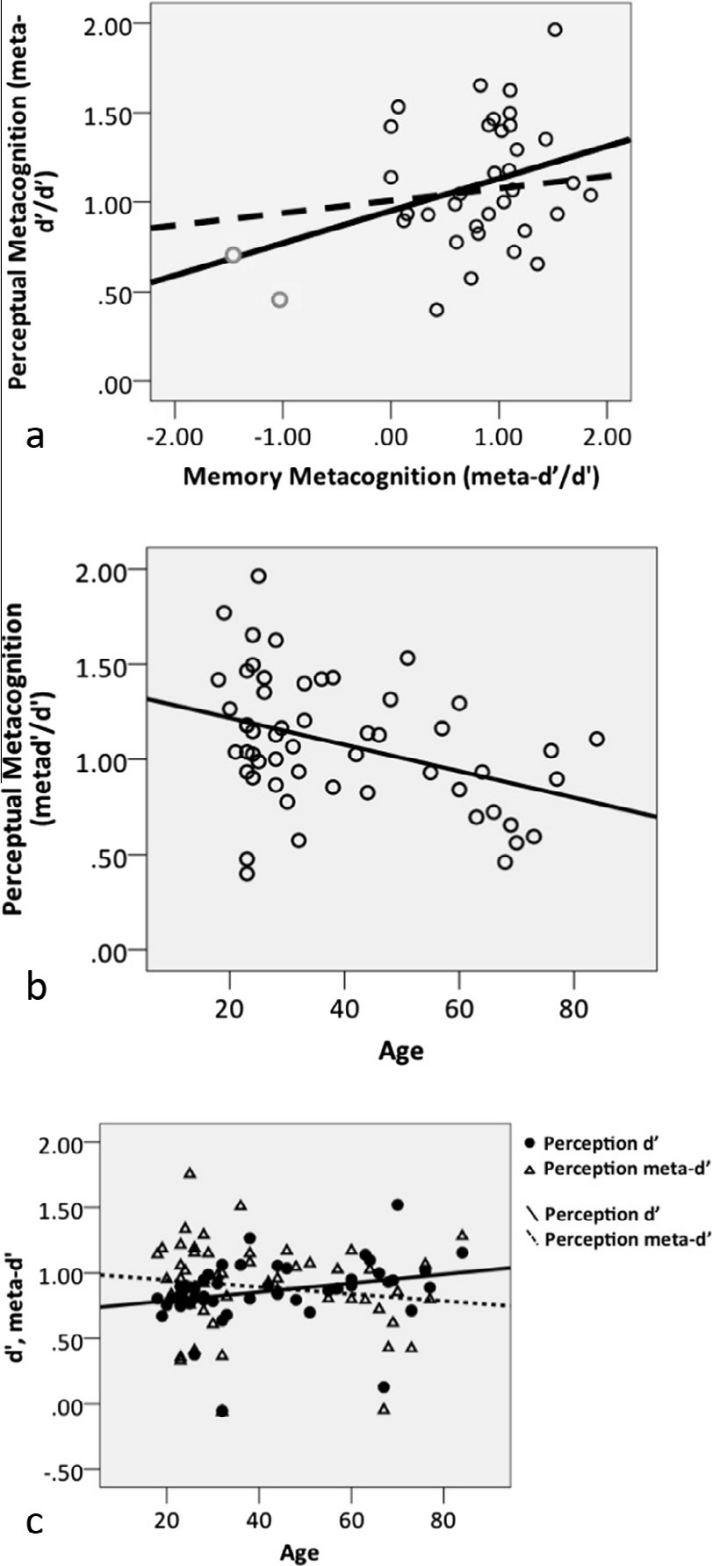
(a) Scatter plot demonstrating the positive relationship between perceptual and memory metacognitive efficiency in the subset of subjects who completed both tasks (*r* = 0.40, *p* = 0.02; *N* = 32). Two outliers are highlighted in grey . The dotted trend line indicates the relationship when outliers are removed (*r* = 0.25, *p* = 0.19; *n* = 30). (b) Scatter plot demonstrating the negative relationship between perceptual metacognitive efficiency and age (*r* = −0.38, *p* = 0.005). (c) Individual components of metacognitive efficiency (d′*,* meta-*d*′) plotted as a function of age. A combined increase in *d*′ and decrease in meta-*d*′ led to the overall decrease in efficiency shown in panel (b).

We calculated a metacognitive efficiency score (meta-d′/d′) for each participant on both perceptual and memory tasks. The average efficiency score was 1.08 (SD = 0.34) for the perceptual task and 0.73 (SD = 0.81) for the memory task. In the subset of subjects who completed both tasks (*N* = 32), metacognitive efficiency tended to be greater for perception than for memory (*t*(31) = 2.19, *p* = 0.04). Consistent with previous findings (McCurdy et al., 2013) we found a positive association between metacognitive efficiencies across domains (*r* = 0.40, *p* = 0.02; *n* = 32; see Fig. 2a.) However, when two memory metacognition outliers were removed (greater than 2 standard deviations beyond the group mean; see Fig. 2a) this relationship failed to reach significance (*r* = 0.25, *p* = 0.19; *n* = 30).

No significant relationships were identified between IQ and metacognitive efficiency (perceptual: *r* = 0.12, *p* = 0.51, *n* = 33; memory: *r* = 0.11, *p* = 0.60, *n* = 27) or years of education and metacognitive efficiency (perceptual: *r* = 0.016, *p* = 0.25, *n* = 53; memory: *r* = 0.003, *p* = 0.98, *n* = 38), consistent with previous reports of a lack of correlation between IQ and metacognition in younger adults (Fleming et al., 2012, Weil et al., 2013). Significant relationships were identified between WMS scores and memory metacognitive efficiency (immediate, *r* = .37, *p* = .05, *n* = 28; delayed, *r* = .59, *p* = .01, *n* = 27; recognition, *r* = .37, *p* = .05, *n* = 27), but not perceptual metacognitive efficiency (immediate, *r* = .12, *p* = .54, *n* = 29; delayed, *r* = .05, *p* = .79, *n* = 28; recognition, *r* = .07, *p* = .73, *n* = 28).

We next turned to the relationship between metacognition and age. We found a significant negative relationship between perceptual metacognitive efficiency (meta-d′/d′) and age (*r* = −0.38, *p* = 0.005; *n* = 53; see Fig. 2b). Thus, despite our measure of metacognition controlling for differences in task performance, older adults showed lower awareness of their perceptual task performance than younger adults. The relationship between age and memory metacognitive efficiency (meta-d′/d′) was negative but was non-significant (*r* = −0.064, *p* = 0.70; *n* = 38). We cannot however draw conclusions regarding a differential effect of age on perceptual compared to memory metacognition as the difference between the domain-specific metacognitive efficiency-age correlations in the subset of subjects who completed both perceptual and memory tasks was itself not significant (Hotelling’s *t* = 0.66, *p* = 0.51).

As meta-d′/d′ is a ratio between two quantities, changes in either or both of its components may contribute to an overall decrease in metacognitive efficiency in the perceptual task. Examining each component separately, in the perceptual task we found a significant increase in d′ with age (*r* = 0.47, *p* < 0.001; *n* = 53) and a non-significant decrease in meta-d′ (*r* = −0.17, *p* = 0.22; *n* = 53). Under the ideal observer model, d′ should be equal to meta-d′. Thus both an increase in performance and a decrease in the ability to appraise this performance (given a particular level of d′) contributed to the observed decrease in overall efficiency (Fig. 2c). For the memory task, there were no significant changes in either d′ (*r* = 0.016, *p* = 0.92; *n* = 38) or meta-d′ (*r* = −0.15, *p* = 0.36) with age.

Finally, we considered that the relationship between metacognitive efficiency and age may be mediated by changes in executive function. In order to assess this hypothesis we constructed a general linear model (GLM) that predicted metacognitive efficiency from age and executive function as measured by the Trail Making Test. This analysis was restricted to a subset of participants who conducted the Trail Making Test (*N* = 44). We found that the relationship between age and perceptual metacognitive efficiency remained significant after controlling for changes in executive function (*β* = −0.0058, *p* = 0.02), and increased executive function was not associated with better metacognitive efficiency (*β* = 0.0042, *p* = 0.26).

## 4 Discussion

The primary aim of this study was to investigate the effects of age on metacognitive efficiency in healthy adults between the ages of 18 and 84. We found that perceptual metacognitive efficiency declined with age, despite task performance being controlled to ensure all participants performed with the same accuracy. In other words, older adults were less efficient at introspecting about whether they are performing well or badly on a perceptual task than younger adults. This result is consistent with previous observations of a weaker match between beliefs and abilities in older adults (Hultsch et al., 2000, Ross et al., 2012) and age-related differences in the accuracy of confidence judgments (Bender and Raz, 2012, Dodson et al., 2007, Huff et al., 2011, Kelley and Sahakyan, 2003, Pansky et al., 2009; Audrey Perrotin et al., 2006, Soderstrom et al., 2012, Souchay et al., 2000, Souchay et al., 2007, Toth et al., 2011, Wong et al., 2012). However, many previous studies did not control for the influence of task performance on measures of metacognition. This is particularly critical when studying aging as metacognitive ability may be difficult to distil from other age-related changes in cognitive abilities. In the current study we employ a measure of metacognition, meta-d′/d′, that controls for the influence of task performance and response bias (Maniscalco & Lau, 2012).

Our results extend those of Weil et al. (2013) who took a similar approach to study the development of metacognitive efficiency during adolescence. In a sample of 28 adolescents and 28 adults it was found that perceptual metacognitive efficiency increased with age during adolescence (11–18 years old), with a non-significant decrease with age in adulthood. However the maximum age in Weil et al’s sample of older adults was 41, precluding the study of metacognitive efficiency in older age. Here we extend this age range to 84, finding that efficiency continues to decline despite task performance remaining stable.

A subset of subjects additionally completed a recognition memory task that allowed us to calculate a metacognitive efficiency score in the memory domain. We found a positive correlation in efficiency scores across domains that was weakened after removal of two outliers. Our result provides some support for the notion that there is a global correlation in metacognitive ability across domains (McCurdy et al., 2013), but also is consistent with a large proportion of domain-specific variance that may be linked to separate brain systems underpinning perceptual and memory metacognition (Baird et al., 2013, McCurdy et al., 2013). There was no effect of age on memory metacognition, but we are cautious about over-interpreting this null result for two reasons: first, fewer subjects completed the memory task, reducing our power to detect an effect of age; and second, there was no statistical support for a differential effect of age on perceptual vs. memory metacognition. Further work is required to ascertain whether may be different trajectories for age-related changes in domain-specific metacognitive functions.

Mixed results were obtained for the effect of age on measures of basic task performance (% correct and d′). In the perceptual task, effects of age on these two measures had opposite sign (negative for% correct and positive for d′). Dissociations between% correct and d′ are possible if the decision criterion is also changing (Macmillan & Creelman, 2004). However such differences in the current study are difficult to interpret, because performance was controlled in the perceptual task such that% correct varied over a narrow range, and poorly performing subjects were excluded prior to analysis. It is possible that increases in d′ with age reflect an overcompensation in difficulty adjustment that made the task slightly easier for older adults. In the memory task, % correct declined with age, whereas this effect was not seen in an analysis of d′.

The meta-d′ approach estimates the subject’s metacognitive accuracy in units directly comparable to d′, a measure of primary task performance (Maniscalco & Lau, 2012). For a metacognitively ideal observer, meta-d′ = d′, and meta-d′/d′ = 1. Closer examination of the relationship between these quantities and age revealed an increase in perceptual d′ and a decrease in perceptual meta-d′, leading to an overall decrease in metacognitive efficiency (Fig. 2c). As noted above, the narrow range of performance levels in the perceptual task precludes strong interpretation of changes in d′. However, the relative values of d′ and meta-d′ are informative. In younger adults, meta-d′ is similar to or slightly above d′ (meta-d′/d′ ∼ 1), whereas in older adults, meta-d′ drops below d′, leading to metacognitive efficiency scores less than expected on an ideal observer model.

One might expect that age-related changes in metacognitive processes would be related to neuropsychological measures of executive function (Fernandez-Duque et al., 2000), following evidence that perceptual metacognitive efficiency is linked to frontal lobe function (Fleming et al., 2010, Hoerold et al., 2013, Persaud et al., 2011, Rounis et al., 2010). Previous studies have found evidence for a link between the use of metacognitive information in the control of behaviour and executive function as measured by Wisconsin Card Sorting Test performance (Pansky et al., 2009, Souchay and Isingrini, 2004), but the relationship between executive function and metacognitive monitoring is less well understood. In particular, it is difficult to rule out an indirect effect of executive function on measures of metacognition via effects on primary task performance, highlighting the need to distil a measure of metacognitive efficiency that controls for differences in performance. In the current study, we did not find evidence for a relationship between perceptual metacognitive efficiency and performance on the Trail Making Test, a measure of set-shifting. Indeed, in a regression analysis explicitly controlling for changes in set-shifting performance, we observed an annual decline of 0.6% in metacognitive efficiency. Of course, executive function is a very broad construct, and the relationship between metacognitive efficiency and other components such as inhibition and updating (Miyake et al., 2000) remain to be determined.

The perceptual and memory metacognition tasks were adapted from those used in recent structural and functional imaging studies of healthy participants (Baird et al., 2013, Fleming et al., 2010, McCurdy et al., 2013). However a limitation of this design is that there are differences between tasks that are potentially orthogonal to the domain in question. For example, our recognition memory task involved verbal stimuli; we cannot rule out the possibility that a different pattern of results would be obtained with memory tasks using nonverbal stimuli such as faces. Indeed, an important goal for future work is the development of perceptual and memory metacognition paradigms that are more closely matched for stimulus characteristics. Similarly it is unclear whether the observed decrease in metacognitive efficiency is specific to visual perceptual metacognition, or may extend to other perceptual modalities or other aspects of decision-making. An additional difference between domains is that the perceptual task used a staircase procedure to control task difficulty for each individual participant whereas this was not feasible with the current word recognition task. By adjusting stimulus selection online such performance control could be built into a memory paradigm in future studies. Finally, a small minority of older individuals struggled to input confidence ratings manually using the keyboard response, and their verbal ratings were instead entered by the experimenter. We cannot rule out effects of changes in response modality on our results, but we note that even in older individuals metacognitive efficiency did not decline to floor levels (meta-d′/d′ ∼ 0), indicating that confidence ratings tracked task performance in a meaningful way regardless of adjustments in response modality.

In summary, we reveal an age-related decline in perceptual metacognitive efficiency whilst controlling for age-related differences in task performance and executive function. Our study is cross-sectional, and it is possible that other factors differing across the age range affected metacognitive ability. It will therefore be very informative in further work to investigate longitudinal changes in metacognitive efficiency with age. Quantifying changes in metacognition with age is critical for our understanding of higher-order cognitive functions in an aging population, especially as deficits in metacognitive monitoring may lead to impaired control of behaviour (Koriat & Goldsmith, 1996). Aging-associated diseases such as Alzheimer’s are accompanied by metacognitive deficits that may lead to non-adherence to treatment and impaired decision-making (Cosentino, 2014). Our results, when combined with previous research in adolescents (Weil et al., 2013), reveal a non-linear relationship between age and perceptual metacognitive efficiency, increasing during adolescence, plateauing in early adulthood, and declining in older age.

## Author contributions

E.C.P, A.S.D and S.M.F. designed research; E.C.P performed research; E.C.P. and S.M.F. analysed data; E.C.P, A.S.D and S.M.F. wrote the paper.
